# Homework Involvement and Academic Achievement of Native and Immigrant Students

**DOI:** 10.3389/fpsyg.2016.01517

**Published:** 2016-10-04

**Authors:** Natalia Suárez, Bibiana Regueiro, Joyce L. Epstein, Isabel Piñeiro, Sara M. Díaz, Antonio Valle

**Affiliations:** ^1^Department of Psychology, University of OviedoOviedo, Spain; ^2^Department of Developmental and Educational Psychology, University of A CoruñaA Coruña, Spain; ^3^Center on School, Family and Community Partnerships, Johns Hopkins University, BaltimoreMD, USA

**Keywords:** immigrants, homework, homework involvement, quality of homework, academic achievement

## Abstract

Homework is a debated issue in society and its relationship with academic achievement has been deeply studied in the last years. Nowadays, schools are multicultural stages in which students from different cultures and ethnicities work together. In this sense, the present study aims to compare homework involvement and academic achievement in a sample of native and immigrant students, as well as to study immigrant students’ relationship between homework involvement and Math achievement. The sample included 1328 students, 10–16 years old from Spanish families (85.6%) or immigrant students or students of immigrant origin (14.4%) from South America, Europe, Africa, and Asia. The study was developed considering three informants: elementary and secondary students, their parents and their teachers. Results showed higher involvement in homework in native students than in immigrant. Between immigrants students, those who are more involved in homework have better academic achievement in Math at secondary grades. There weren’t found gender differences on homework involvement, but age differences were reported. Immigrant students are less involved in homework at secondary grades that students in elementary grades. The study highlights the relevance of homework involvement in academic achievement in immigrant students.

## Introduction

Schools are complex places in which students of many races, ethnicities, cultures, religions, and economic conditions work together. They and their families bring many characteristics to school that provide opportunities to enrich student learning every day. The diversity of students is increasing in schools in many countries. In the last decade, immigration has been an important and socially debated issue. Particularly, in the US, more than 40% of all public school students are from diverse cultures, doubling the percentage of 1980s (Hutchins et al., 2012). One in five children in the US has at least one foreign-born parent (Hernandez et al., 2007). In Spain, in the last 20 years, the number of immigrants has increased considerably. In 1998, there were 500,000 people, 1.6% of the total Spanish population. That number has increased to 4.5 million people in 2007, 10% of the total Spanish population (Encuesta Nacional de Inmigrantes, 2007).

At school, as in society, immigrant students live and work side by side with native-born students. Immigration can be a stressful event which brings changes to the family system (Suárez-Orozco and Suárez-Orozco, 2001). Some immigrant families are well educated and their children achieve high levels in school, whereas other families are unfamiliar with schools and educational requirements, and often have children in under-resourced schools. These students tend to experience greater stress in school, and are less likely to graduate from high school or attend and complete college (Cooper et al., 2006).

Some studies have reported that, on average, students from immigrant families lag behind other students in reading, writing, math, science, and other subjects (Bang et al., 2009). However, other researchers have report that there is more diversity in achievement within groups than between groups of immigrant students (De Jong et al., 2000; Cooper and Valentine, 2001). Variations in student achievement may depend on pre-immigration factors, such as family income and parents’ education levels (Núñez et al., 2014).

Despite racial or ethnic background, families differ in their beliefs, values, needs, and resources (Trautwein, 2007; Tam, 2009). It is well known that socio-economic status is one of the most important demographic factors related to children’s development and learning. Parents who are employed and in higher-paying jobs are more capable of providing the educational resources and support children needs (Bang et al., 2009).

Other variables also affect student success in school. Students’ attitude toward schoolwork and homework are associated with achievement. Homework patterns have been extensively studied in students in general, but few studies have examined immigrant students’ investments in homework. Their home environments should be taken into account to understand immigrant students’ homework behaviors and their parents’ involvement in students’ homework. This study addresses this gap in knowledge about the homework process with analyses of selected variables regarding homework in native and immigrant boys and girls.

### Homework and Academic Achievement

Homework assignments have multiple purposes. Some are instructional but others have behavioral goals. Teachers may assign some homework to enable students to practice specific skills, but they also may aim to help students develop their responsibility, perseverance, and time management (Epstein and Van Voorhis, 2001). Teachers also assign homework to assess the extent to which students have mastered specific skills in order to plan new lessons that will meet students learning levels (Bang et al., 2009; Rodríguez et al., 2014).

Over past few decades, many studies of homework processes have been conducted, mainly to clarify the importance of homework for students’ academic achievement (Valle et al., 2015b).

Prior studies have raised several issues that need more attention in new research. We need to better understand such questions as: *What is the relationship between homework and students’ academic achievement? Is the quantity of homework completed a good predictor of academic achievement? Are time spent on homework and homework time management important indicators of student learning?* Different studies have reported a variety of results to these questions.

About homework completion, the amount of homework that students complete seems to be positively related to academic achievement (Cooper et al., 1998; Cooper et al., 2001; Cooper et al., 2006; Núñez et al., 2013, 2015). However, there are significant discrepancies about the relationship between time spent on homework and academic achievement. Some studies report positive connections, suggesting the more time spent on homework, the higher students’ academic achievement (Cooper, 1989; Walberg, 1991; Cooper and Valentine, 2001; Cooper et al., 2006). Others report that relationship is weak or negative (De Jong et al., 2000; Trautwein et al., 2002, 2006, 2009; Trautwein, 2007; Tam, 2009; Fernández-Alonso et al., 2014). Xu (2007) explained a null relationship by noting that when students spend more time on homework they may not be managing homework efficiently. In fact, students’ homework time management also determines the quantity of homework students do from those assigned by their teachers even better that time spent on homework (Regueiro et al., 2014). Recent studies reinforce that finding. Núñez et al. (2013) reported that homework time management was a crucial variable for determining students’ academic achievement—more important than the quantity of homework completed or the quantity of time spent doing homework—. Another study showed that the quantity of homework done was associated with better academic results (Núñez et al., 2015).

Students’ gender and grade level also have been related to homework involvement as important variables. Most studies confirm that girls are more committed to doing homework than boys (Younger and Warrington, 1996; Xu, 2006, 2007; Núñez et al., 2013). Many studies also confirm that homework is more strongly related to academic achievement in high school than in middle and elementary school (Cooper and Valentine, 2001), but other authors maintain that there are interesting reasons for this relationship. For example, some older students are less engaged (Núñez et al., 2013), persisted less and enjoyed less doing homework than their younger colleagues (Hong et al., 2009). Epstein (2011) contends that the connection of homework and achievement at the high school level is exaggerated because some high school students stop doing homework and because teachers give advanced students more homework to complete.

Most studies of homework have been conducted with samples of native students, who complete their work without having to face the difficulties of attending school in a new country in a new educational system, and without having to learn a new language. Presently, in many countries, native and new immigrant students attend school together. The changing populations of students in schools raise important questions about native and immigrant students’ homework behaviors and results of homework for learning outcomes.

### Homework and Immigrant Students

It is common, due to challenges related to relocation in a new country that immigrant students lag behind native students in academic achievement. Homework, which can be completed slowly, thoughtfully, and with assistance, can be one way to close the achievement gap between these groups of students. Homework may provide opportunities for immigrant students to practice and review lessons. Or, homework may disadvantage immigrant students and widen the achievement gap if, due to language difficulties, they are unable to comprehend and complete their assignments (Bang et al., 2009).

Studies of immigrant students’ homework experiences are scarce. One of the few studies on the topic reported that individual characteristics such as student interest, engagement, and learning style were the most important factors associated with immigrant students’ homework completion (Bang, 2011b). This study also found that some measures of family and school environments also contributed to immigrant students’ homework behaviors. For example, students who paid attention in class and followed school rules, recognized that homework would help them perform better in school (Bang et al., 2009), just as native students do (Trautwein, 2007). A study of 9th– 12th grade newcomer immigrant students in the US showed that students who had stronger interest in class and more structured homework environments were more likely to complete their homework than were their less engaged peers (Bang, 2011a). The same study found that students who had received instruction to fully understand their course materials and who attended homework coaching sessions were more likely to do their homework.

These findings echo that of previous research indicating that students who carried out behaviors conducive to academic success were more likely to complete homework than their peers who were less engaged in school (Goslin, 2003; National Research Council, 2004).

Given the links between homework, report card grades, and achievement, it is important to understand the factors that facilitate and impede students’ homework completion in order to support immigrant students’ academic endeavors (Bang et al., 2011).

A study of a sample of recently arrived immigrants to the US, aged 9–14, highlighted the important role of completing homework on grades and achievement (Bang et al., 2009). The authors reported that completing homework was even more important for predicting grades than students’ English language proficiency or teachers’ ratings of their understanding and behavior.

Students’ gender also may be a significant predictor of homework completion by immigrant students (Xu, 2006; Bang et al., 2009). Bang (2011b) showed that in general girls were more likely than boys to complete homework, in line with findings in native students’ samples (Xu, 2006; Núñez et al., 2013; Suárez-Orozco and Qin-Hilliard, 2015).

### The Current Study

The few extant studies on homework patterns of immigrant students (Mau and Lynn, 1999; Bang et al., 2009; Bang, 2011a) identify an important agenda but only roughly explored connections of students’ homework engagement and academic achievement. These studies did not analyze details of the homework process such as the amount of homework completed, the time spent on homework, and the quality of the homework and its relationship with academic achievement.

The present research has three main objectives: (i) to explore, compare, and contrast homework behaviors of immigrant and native-born students in Spain; (ii) to examine relationships of homework engagement (i.e., amount of homework completed, time spent, time management, and quality of homework completed) and benefit from doing homework with immigrant students’ math achievement; (iii) to analyze whether the homework engagement variables and academic achievement are associated with the gender and grade level of immigrant students, as was reported in studies of native student populations.

## Materials and Methods

### Participants

The study involved 1328 students, 10–16 years old (*M* = 13.11, *SD* = 1.75) attending 29 public schools in Spain. The students are from Spanish families (*n* = 1137, 85.6%) or are immigrant students or students of immigrant origin (*n* = 192, 14.4%). The immigrant students came from South America (*n* = 127), Europe (*n* = 54), Africa (*n* = 9), and Asia (*n* = 2).

The sample includes 617 students in the Elementary Grades (i.e., 5th and 6th grade, 487 native, and 130 immigrant students), and 712 students in the Secondary Grades (Compulsory Secondary Education, i.e., grades 1–4, 650 native, 62 immigrant students). The parents of these students also participated as informants as well as one teacher of each class of students.

This study was carried out in accordance with the recommendations of The Declaration of Helsinki. All the subjects gave written informed consent.

### Measures

*Time spent on homework, homework time management, amount of homework completed, quality of homework done*, and *benefit of doing homework*.

To measure these five variables, we used the Homework Survey (e.g., Rosário et al., 2009; Núñez et al., 2013, 2015; Valle et al., 2015a), which is composed of three parts, using three sources of information for some of its variables.

One survey part is answered by students, other survey part by parents and the third one by teachers.

The studied variables had different respondents: students, teachers, and parents were asked about *students’ amount of homework done*; students and parents were asked about *students’ time spent doing homework* and *students’ homework time management*; and teachers were asked about *students’ quality of homework done* and *students’ benefit of doing homework*.

*Amount of homework completed* by students (from the total homework assigned by teachers) was collected considering the three agents and was obtained through responses to an items about the amount of homework usually done, using a 5-point Likert-type scale (1 = none, 2 = some, 3 = one half, 4 = almost all, and 5 = all).

*Time spent on homework* was measured through information provided by parents and students, responding to the item with the general formulation, “How much time do you/the student usually spend on homework?” Response options were: 1 = less than 30 min, 2 = 30 min to 1 h, 3 = 1 h to an hour and a half, 4 = 1 h and a half to 2 h, and 5 = more than 2 h.

*Homework time management* was measured through information provided by parents and students, responding to the item with the general formulation, “How do you/the student manage the time normally spent doing homework?” Response options were: 1 = I waste it completely (I am constantly distracted), 2 = I waste it more than I should, 3 = regular, 4 = I manage it pretty well, and 5 = I optimize it completely (I concentrate and until I finish, I don’t think about anything else). In case of parents’ item, the formulation was the opposite; the item refers to waste of time instead of good time management, “My child wastes time when doing homework”.

In addition to these variables, two variables informed by the teacher were considered: *Quality of homework done* and *Benefit of doing homework for the students*. Both variables were responded by teachers. *Quality of homework done* was measured by the item “How does the student do his homework?” Response options were: 1 = Very good, 2 = Good, 3 = Fair, 4 = Poor, and 5 = Very poor.

*Benefit of doing homework* was measured by the item “Does the student take benefit from doing homework?” Response options were: 1 = Strongly disagree, 2 = Disagree, 3 = Undecided, 4 = Agree, and 5 = Strongly agree.

*Academic Achievement*. Assessment of academic achievement was obtained through students’ report card grades in Mathematics.

### Procedure

The data on the students’ survey were collected in one class period during regular school hours by external staff, after obtaining the consent of the school directors and the students’ teachers. Data on teachers’ survey were collected while students were answering their survey in class. Parents’ survey was sent to home and brought it back to school when done.

Prior to the administration of the surveys, the participants were informed of the importance of responding sincerely to the items. They were told that their reports were confidential and would be used only for research purposes.

### Data Analysis

Taking into account the goals of this study, the data were analyzed with Student’s *t*-test for independent samples to determine differences between native-born students and immigrant students, and to analyze gender differences and differences related to the educational stage. The existence or nonexistence of variance homogeneity was taken into account when interpreting the results. For this was taken as reference value “*p*” in the Levene test, which involves taking the existence of equal variances when *p* > 0.05, whereas when *p* < 0.05 is considered that the variances are not equal. Based on this criterion, the value of “*t*” is also selected. We used Pearson correlational analysis to study the relation between homework-related variables and academic achievement in the sample of immigrant students.

## Results

### Analysis of the Differences between Native and Immigrant Students in the Dependent Variables

**Table 1** shows the descriptive statistics on the homework measures for native-born and immigrant students in the full sample of students, parents, and teachers, and on the achievement measures at the elementary and secondary levels.

**Table 1:** Descriptive statistics on homework measures for native and immigrant students.

	Group	*N*	*M*	*SD*
Student: *How much homework do you do?*	Native	1127	4.31	0.996
	Immigrant	191	4.25	0.887
Student: *Daily homework time Monday to Friday*	Native	1125	3.06	1.122
	Immigrant	191	2.97	1.147
Student: *When doing homework, I concentrate until I finish*	Native	1125	2.80	1.182
	Immigrant	190	2.59	1.103
Parents: *My child wastes time when doing homework*	Native	662	2.51	1.497
	Immigrant	130	2.23	1.626
Parents: *Daily homework time from Monday to Friday*	Native	661	3.04	1.247
	Immigrant	130	2.58	1.589
Parents: *How much homework do you do?*	Native	664	4.59	0.992
	Immigrant	130	3.91	1.815
Primary Mathematics grade	Native	471	4.09	1.691
	Immigrant	129	4.32	2.050
Secondary Mathematics grade	Native	650	5.71	2.345
	Immigrant	61	4.67	2.014
Teachers: *Does he/she do his/her homework?*	Native	1094	3.94	1.156
	Immigrant	191	3.56	1.328
Teachers: *Does homework benefit him/her?*	Native	1092	3.95	1.171
	Immigrant	191	3.65	1.263
Teachers: *How is his/her homework?*	Native	1093	3.80	1.168
	Immigrant	191	3.49	1.252

*Amount of homework done* (of the assigned homework). The data indicate that, although there were no significant differences between native and immigrant students in their own reports about homework completed, *t*(1316) = 0.864, *p* > 0.05, both the parents and the teachers for the two groups of students reported statistically significant differences on homework completed by the children [parents: *t*(144) = 4.150, *p* < 0.001, *d =* 0.58, medium effect size; teachers: *t*(243) = 3.738, *p* < 0.001, *d* = 0.32, small effect size]. Parents and teachers reported that native-born students completed more homework than did immigrant students. The students’ reports were in the same direction, but the differences of native and immigrant students’ reports were not significant.

#### Weekly Homework Time

Native and immigrant students report similar amounts of time spent on homework, but their parents’ reports show a different pattern. Parents of native students report that their children spend more time on homework [(*t*(162) = 3.113, *p* < 0.001, and *d* = 0.35, small effect size].

#### Time Management (Use of Time Dedicated to Homework)

As seen in the data provided in **Table 1**, whereas the native students state that they concentrate more on their homework than the immigrants, *t*(1313) = 2.340, *p* < 0.05, and *d* = 0.18, with a small effect size, the parents of the native students indicate that their children waste more time than that indicated by the parents of immigrant children, *t*(709) = 1.941, *p* < 0.05, and *d* = 0.18, with a small effect size.

#### Quality of Homework Done

The teachers indicate that the quality of the native students’ homework is better than that of the immigrant students, *t*(1282) = 3.369, *p* < 0.001, and *d* = 0.26, with a small effect size.

#### Benefit of Doing Homework

The teachers also think that homework benefits the native students more than the immigrants, *t*(250) = 2.992, *p* < 0.01, and *d* = 0.25, with a small effect size.

#### Academic Achievement (Students’ Grades in Mathematics).

Whereas at the elementary school level, there were no statistically significant differences in mathematical academic achievement between the two groups of students, *t*(178) = -1.174, *p* > 0.05, the groups differed at the Secondary level. Native-born students had significantly higher math scores than immigrant students, *t*(709) = 3.345, *p* < 0.001, and *d* = 0.26, with a small effect size.

### Relation between Homework Engagement and Mathematical Achievement in Immigrant Students

**Table 2** shows the correlations among the variables involved in the study for immigrant students.

**Table 2:** Matrix Zero-order correlations of the variables of the study from immigrant students.

	Gender	Stage/Level	Student	Student	Student	Parents	Parents	Parents	Teacher	Teacher	Teacher	AMP	AMS
			V1	V2	V3	V1	V2	V3	V1	V2	V3		
Gender	1												
Stage	0.025	1											
Student_V1	-0.013	-0.417ˆ**	1										
Student_V2	0.121	-0.093	0.158ˆ*	1									
Student_V3	0.019	0.292ˆ**	-0.244ˆ**	-0.016	1								
Parents_V1	-0.072	0.139	0.025	0.024	0.06	1							
Parents_V2	0.003	0.082	0.102	0.300^∗∗^	-0.054	0.524^∗∗^	1						
Parents_V3	-0.029	0.109	0.145	0.031	-0.087	0.533^∗∗^	0.704^∗∗^	1					
Teachers_V1	0.031	-0.190ˆ**	0.323ˆ**	0.112	-0.115	0.021	0.357^∗∗^	0.457^∗∗^	1				
Teachers_V2	0.062	-0.176ˆ*	0.299ˆ**	0.094	-0.101	0.097	0.343^∗∗^	0.437^∗∗^	0.743^∗∗^	1			
Teachers_V3	0.014	-0.070	0.258ˆ**	0.136	-0.127	0.083	0.320^∗∗^	0.413^∗∗^	0.831^∗∗^	0.787^∗∗^	1		
AMP	0.009	-0.017	0.167	0.054	-0.162	-0.258^∗∗^	-0.133	-0.139	0.323^∗∗^	0.195^∗^	0.371^∗∗^	1	
AMS	-0.024	-0.180	0.355ˆ**	0.100	0.041	0.224	0.243	0.442	0.267^∗^	0.21	0.248	a	1

*The sizes of the correlations are not directly comparable because the sample sizes are different in many cases. Gender (1 = Male, 2 = Female); Stage (1 = Elementary, 2 = Secondary); Student_V1 (amount of homework done, informed by students); Student_V2 (homework time, informed by students); Student_V3 (time management, informed by students); Parents_V1 (waste of time, informed by parents); Parents_V2 (homework time, informed by parents); Parents_V3 (amount of homework done, informed by parents); Teachers_V1 (amount of homework done, informed by teachers); Teachers_V2 (benefit from homework, informed by teachers); Teachers_V3 (quality of homework done, informed by teachers); AMP (academic achievement in mathematics in Elementary Education); AMS (academic achievement in mathematics in Secondary Education). *N* = 192. ^∗∗^*p* < 0.01 and ^∗^*p* < 0.05.*

*^a^Correlation between AMP and AMS is not studied because there are two different samples (secondary and elementary students).*

The results obtained indicated that the amount of homework done positively correlated with academic achievement in mathematics, according to the students, at secondary level (*r* = 0.355, *p* < 0.01, and *d* = 0.34, small effect size) and the teachers, at elementary and secondary levels (*r* = 0.267, *p* < 0.05, and *d* = 0.73, medium effect size). The relation between homework quality and mathematical achievement was also statistically significant, albeit at a lower level (*r* = 0.248, *p* = 0.054, and *d* = 0.81, large effect size). Neither time dedicated to doing homework nor time management had a significant association with immigrant students’ mathematical achievement.

In general, there were no significant connections of gender and any homework-related variables or math achievement.

In terms of parents’ reports, they are more consistent with teachers’ reports than with their own students’ reports. Parents’ and students’ reports are only consistent regarding time spent on homework (*r* = 0.300 and *p* < 0.01). But, parents’ *homework time* report and *amount of homework done* report are strongly associated with all teachers’ reports (*amount of homework done, quality of homework*, and *benefit from homework*).

### Gender and Educational Stage Differences in Immigrant Students’ Homework Engagement

Results obtained after performing the corresponding analysis of differences (through Student’s *t*) showed that the gender of the immigrant students was not significantly associated with any of the dependent variables.

**Table 3** presents data on immigrant students’ homework behaviors by educational level: elementary grades (5th and 6th) and secondary grades (1st to 4th grade of Compulsory Secondary Education).

**Table 3:** Descriptive statistics by school level (elementary/secondary) for immigrant students.

	School Level	*N*	*M*	*SD*
Student: Amount of homework done	Elementary	129	4.43	0.748
	Secondary	62	3.85	1.022
Student: Weekly homework time	Elementary	129	3.01	1.169
	Secondary	62	2.89	1.103
Student: Time management	Primary	128	2.32	1.057
	Secondary	62	3.15	0.989
Parents: Waste of time	Primary	116	2.11	1.592
	Secondary	14	3.21	1.626
Parents: Weekly homework time	Primary	116	2.53	1.618
	Secondary	14	3.00	1.301
Parents: Amount of homework done	Primary	116	3.84	1.891
	Secondary	14	4.43	0.852
Teachers: Amount of homework done	Primary	129	3.71	1.325
	Secondary	62	3.26	1.292
Teachers: Benefit obtained	Primary	129	3.81	1.269
	Secondary	62	3.34	1.200
Teachers: Quality of homework	Primary	129	3.53	1.293
	Secondary	62	3.42	1.167

Students and teachers informed of a greater amount of homework done in Primary compared to Secondary Education [students: *t*(189) = 4.429, *p* < 0.001, and *d* = 1, large effect size; teachers: *t*(189) = 2.202, *p* < 0.05, and *d* = 0.34, small effect size], although the parents indicated that the differences favor Secondary Education, *t*(31) = -2.031, *p* = 0.051, and *d* = 0.33, with a small effect size. Likewise, students and parents informed of statistically significant differences in homework time optimization [students: *t*(188) = -5.148, *p* < 0.001, and *d* = 0.81, large effect size; parents: *t*(128) = -2.441, *p* < 0.05, and *d* = 0.69, medium effect size] but in the opposite direction: whereas secondary students believed they make better use of their time, their parents thought they waste time the most. Lastly, the teachers thought that, although there were no statistically significant differences with regard to quality by educational stage, doing homework benefits Primary students more, *t*(189) = 2.425, *p* < 0.05, and *d* = 0.38, with a small effect size.

## Discussion

The main goal of this research was to shed some light on the influence of doing homework on the academic achievement of immigrant students in Spain.

The study aimed to extend general patterns of the value of homework reported in prior investigations (Mau and Lynn, 1999; Bang et al., 2009; Bang, 2011a). The study also benefited from unique data on homework behaviors from native and immigrant students in Spanish elementary and secondary grades.

General findings highlight that:

1. When comparing native and immigrant student, the full sample in this study indicated that on all measure of homework (time, time management, and homework done) and on Math achievement, student, parent, and teacher mean scores were slightly higher for native students than for immigrant students.
2. When focusing only on immigrant students, all homework measures from all reporters (immigrant students, parents, and their teachers) were positive correlated with student achievement at the secondary level. In particular, there were significant correlations of math achievement with student and teacher reports about the immigrant students’ homework completion.
3. These results suggest that among immigrant students, those who take the role of student seriously and do their homework have higher math achievement than immigrant students who do not complete their assignments. Independent reports from students and from teachers indicate that doing homework benefits immigrant students, much as prior studies report doing homework benefits other students (mainly native students in prior studies).
4. There were no significant patterns of gender differences among immigrant students on these measures.
5. Statistically significant differences were found as a function of grade in immigrant students (**Table 3**). As they progress through the grades, older immigrant students report lower mean scores for the amount of homework done, time on homework, and time management while doing homework. Teachers report similar patterns that immigrant students at the secondary level do less homework, do lower quality work, and benefit less from homework than do students at the elementary level.

This study contributed new ideas for studies of the homework patterns of immigrant students of Spain or other countries. It was possible, then to contrast patterns of homework behavior for native-born and immigrant students, and then, within the immigrant student sample, the implications of doing homework on student achievement.

Summing up, this study strongly suggests that doing homework is beneficial for immigrant students, although, presently, they involve less in homework and lag on achievement compared to native-born students.

The data were particularly interesting because they included reports from students, parents, and teachers about immigrant students’ homework behaviors, and included other useful data on students’ Math achievement. Parents’ reports differenced from student and teacher reports about students at the elementary and secondary levels. Parents reported that they observed that their secondary students spent more time, and completed more homework than students in the elementary grades, but secondary students waste more the time spent on homework that elementary students. Students and teachers disagree and they consider elementary students do more homework and spend more time than secondary students but their time management is worse than in higher levels. Other studies considered that secondary students were less engaged and enjoyed less doing homework (Hong et al., 2009; Núñez et al., 2013) where the informants were the students.

Results indicated that there were no significant differences of immigrant male and female students on the detailed homework variables. These results contrast with prior studies of both native (Núñez et al., 2015) and immigrant populations (Trautwein, 2007; Bang, 2011a), that typically indicate that female students do more and better on homework. Future studies with larger populations of immigrant groups may reveal clearer patterns of homework behavior of male and female students. There may be some clues about this, as noted by Bang et al. (2011). For example, immigrant families may have fewer financial resources, and may demand more help from their children with housework, such as caring for younger siblings. This may leave students with less time for doing homework, or reduce girls’ advantages in doing more school work and make their patterns of homework similar to boys.

### Limitations and Future Research

There still were some inconsistent results of this study that will need attention in future research. In particular, larger samples of immigrant students at specific grade levels are needed. Information also is needed on how long the students have been in a country so that researchers can account for those who are becoming assimilated compared to those whose families have been settled for several generations.

Several variables have been pointed out that can affect student engagement in school and on homework. These include: mastery of the language (Bang et al., 2011); the culture of origin (Keith et al., 1998; Mau and Lynn, 1999); the help offered by the parents (Sibley and Dearing, 2014; Núñez et al., 2015; Madjar et al., 2016); the feedback given by the teacher (Núñez et al., 2014; Rosário et al., 2015).

Also, the type of homework assigned to the students should also be examined, because not every kind of homework is equally effective for improving subject specific academic achievement.

In this study, the data were exploratory due to the size and composition of the immigrant student sample. Future studies with larger and more coherent samples of students from the same continent or country of origin will be able to establish a predictive model to isolate the independent effects and mediating effects of particular background, grade level, and homework variables on student achievement.

## Author Contributions

All authors have contributed to this study, collecting the sample, doing the data analysis and writing and discussing the results.

## Conflict of Interest Statement

The authors declare that the research was conducted in the absence of any commercial or financial relationships that could be construed as a potential conflict of interest.
